# How to Breathe

**Published:** 1897-04

**Authors:** 


### SELECTIONS.

### How to Breathe.

It is of the utmost importance to accustom one's self when walking to frequent intervals of conscious breathing. No involuntary action of the body is habitually so carelessly performed—so almost shirked—as this one, and upon no other does our health so largely depend. The great majority of the human race keep their lungs in a state of semi-starvation; and diseases and ailments manifold can be traced to this cause alone, since the very act which deprives one of life-giving oxygen also returns to the arteries impure blood, weighted with poisonous carbonic acid.

If the lungs be properly inflated, this act alone gives the body a buoyancy, which increases the pleasure, and lessens the exertion, of walking. Of course a mincing or languid step must be avoided. Take a free and firm, but light stride, balancing the upper part of the body alternately on each hip—but without swaying it perceptibly—and giving the impetus forward with a slight spring from the ball of the foot. Naturally, the mind will at first have to direct these motions, but the body responds delightfully to right ways of doing things, and if the exercise of walking can be taken where there is much of interest to divert one, it will be found a great advantage, for this ready and cheerful response of the entire body, when its muscles are thus called into harmonious action, imparts a sense of exhilaration which makes one feel more like a bird than anything else can until flying-machines have become accomplished facts.

The lungs have their own muscular power, which, unfortunately, is not more than half developed. The simplest preparatory exercise is full, deep breathing. Draw in a long, deep breath, expanding the chest as fully as possible without straining either lungs or muscles. Retain the breath thus taken while you count ten; then, as slowly as possible, expel it. This conscious breathing will soon enlarge and strengthen the lungs, and the more frequently this conscious action can be made, the better for the lungs and health.

Remember in all breathing exercises that nature's avenue to the lungs is through the nostrils; provision is made in the nasal passages to catch impurities and foreign substances, which, if carried to the lungs, as when breathing through the mouth, are liable to cause serious trouble. The very best time to practice lung gymnastics is in the morning before dressing, and again at night, for the body should be free from all restraining clothing.

Stand erect, with chin down, and rise upon the toes as you inhale; hold the breath for a few moments, so that the air may act on the whole surface of the blood, nourishing it and at the same time taking up impure gases; then expel it forcefully and as completely as possible, coming down on the heels at the same time. Five minutes of this work night and morning will work wonders.

If a proper carriage of the body be retained in all the ordinary duties of life, whether sitting or walking, it will be found to greatly minimize the fatigue of daily duties. It is the throwing of double work on some muscles by leaving others in idleness that causes more than half the pain of back and limbs which women suffer. If you will walk up stairs properly, with figure erect, legs and joints flexible, breathe properly, it is a healthful exercise, which can not harm even a feeble woman.—MARIA DUNCAN, M. D., *in Progressive Age.*

